# Tuméfaction appendue au mamelon

**DOI:** 10.11604/pamj.2014.18.227.4847

**Published:** 2014-07-18

**Authors:** Ahlam Abdou, Badreddine Hassam

**Affiliations:** 1 Service de Dermatologie, CHU Ibn Sina, Université Med V, Souissi, Rabat, Maroc

**Keywords:** Molluscum pendulum, acrochordon, mamelon, papillomatose, Molluscum pendulum, acrochordon, nipple, papillomatosis

## Image en medicine

Le molluscum pendulum, ou acrochordon, ou polype fibroépithélial est une formation tumorale cutanée bénigne qui prend la forme d'une excroissance pédiculée. Ces lésions sont généralement petites, molles, rosées ou hyperpigmentées. Ce sont des polypes qui mesurent moins de 1 mm à plus de 1 cm (rarement). Elles se localisent aux grands plis, mais également parfois aux paupières. Leur nombre augmente avec l’âge. La base étroite est évocatrice. Leur origine est mal comprise. Elles ont pour origine un dérèglement de la reproduction des cellules des glandes sébacées hypertrophiées. Si les lésions sont asymptomatiques, aucun traitement n'est nécessaire sauf si elles montrent des signes d'irritation par frottement notamment ou pour des raisons esthétiques. Le diagnostic différentiel se fait avec les naevi naevo-cellulaires dermiques “mous” (qui ont généralement une base plus large), les kératoses séborrheïques pédiculées et les papillomes verruqueux. Le traitement est basé sur l'excision chirurgicale. Nous rapportons l'observation de madame M.A. âgée de 29 ans sans antécédents pathologiques notables qui présente depuis 6 ans une lésion appendue au mamelon du sein droit qui a augmenté progressivement de taille faisant corps avec celui-ci constituée de papules brillantes en grappe lisses ayant la même couleur du mamelon et indolore. Nous avons évoqué devant cette excroissance un mamelon surnuméraire, un molluscum pendulum, hyperkératose mammelonaire et un botriomycome épidermisé. Une électrosection a été indiquée. L'examen histologique a mis en évidence une lésion papillomateuse dont le revêtement malpighien est discrètement kératosique reposant sur un axe fibreux peu vasculaire en faveur d'un fibrome mou.

**Figure 1:**
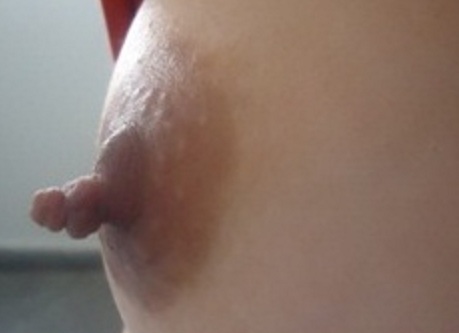
Tuméfaction appendue au mamelon

